# Activation of ER Stress-Dependent miR-216b Has a Critical Role in *Salvia* *miltiorrhiza* Ethanol-Extract-Induced Apoptosis in U266 and U937 Cells

**DOI:** 10.3390/ijms19041240

**Published:** 2018-04-19

**Authors:** Changmin Kim, Hyo-Sook Song, Hojung Park, Bonglee Kim

**Affiliations:** 1 Department of Pathology, College of Korean Medicine, Graduate School, Kyung Hee University, 1 Hoegi-dong, Dongdaemun-gu, Seoul 130-701, Korea; ckdals4302@khu.ac.kr (C.K.); rosapark93@khu.ac.kr (H.P.); 2 Department of Science in Korean Medicine, College of Korean Medicine, Graduate School, Kyung Hee University, 1 Hoegi-dong, Dongdaemun-gu, Seoul 130-701, Korea; shs331@khu.ac.kr

**Keywords:** *Salvia miltiorrhiza*, oxidative stress, endoplasmic reticulum stress, C/EBP homologous protein, reactive oxygen species, miR-216b, c-Jun

## Abstract

Although *Salvia* *miltiorrhiza* has been reported to have anti-cancer mechanisms, such as caspase activation, cell cycle arrest, an anti-angiogenesis effect, and Bcl-2 family regulation, its underlying mechanism of endoplasmic reticulum (ER) stress-mediated apoptosis has never been demonstrated. Thus, in this current study, ER stress-related apoptosis via miR-216b of the ethanol extract of *Salvia* *miltiorrhiza* (SM) is elucidated for the first time. SM treatment inhibited the viability of U266 and U937 cells in a concentration-dependent manner. However, SM-exposed Raw264.7 cells were intact compared to U266 or U937 cells. Treatment with SM significantly elevated the generation of reactive oxygen species (ROS). The anti-proliferative effect of SM was reversed by pretreatment with the ROS scavenger, *N*-acetyl-l-cysteine (NAC), compared to cells treated only with SM. Also, SM treatment increased the ER stress by elevation of phosphorylated activating transcription factor 4 (p-ATF4), phosphorylated eukaryotic Initiation Factor 2 (p-eIF2), and phosphorylated protein kinase RNA-like endoplasmic reticulum kinase (p-PERK) expression. Caspase-3 and Poly (ADP-ribose) polymerase (PARP) were cleaved and CCAAT-enhancer-binding protein homologous protein (CHOP) was activated by SM treatment. PARP cleavage and CHOP activation were attenuated by NAC pretreatment. Furthermore, SM increased the tumor suppressor, miR-216b, and suppressed its target, c-Jun. miR-216b inhibitor attenuated the apoptotic effect of SM. Taken together, SM treatment induced apoptosis through regulation of miR-216b and ROS/ER stress pathways. SM could be a potential drug for treatment of multiple myeloma and myeloid leukemia.

## 1. Introduction

Myeloid-originated hematological malignancies, including multiple myeloma and myeloid leukemia, have been continuously increasing worldwide [1,2]. Multiple myeloma, known as plasma cell myeloma, arises through the expansion of malignant plasma cells derived from B cells in bone marrow. Patients with multiple myeloma often suffer from hypercalcemia, fractures, renal insufficiency, and neuropathy [3,4]. Human myeloid leukemia represents an increase in the number of myeloid cells and their abnormal differentiation in the bone marrow, leading to hematopoietic insufficiency. Patients diagnosed with human myeloid leukemia have symptoms of fatigue, hemorrhage, infection, and organ infiltration, such as hepatomegaly and splenomegaly [5,6,7,8].

Myeloid-originated hematological malignancies are able to escape programmed cell death or suppression of proliferation and metastasis. Therefore, induction of apoptosis has been regarded as a target for cancer treatment [9,10,11,12]. Normally, apoptosis is implemented by intrinsic and extrinsic pathways. Recent studies have suggested that endoplasmic reticulum (ER) stress mediates apoptosis in cancer cells [13,14]. Furthermore, several studies relevant to ER stress-mediated apoptosis in myeloid-originated hematological malignancies have been reported [8,15,16].

ER provides a principal site for functional protein folding and synthesis. However, the protein-folding machinery in ER is challenged by oxidative stress, hypoxia, or the low potential of hydrogen (pH) [17,18]. In particular, reactive oxygen species (ROS) promote the accumulation of misfolded protein in ER, which is termed “ER stress”, by damaging calcium (Ca^2+^) homeostasis in ER lumen [19]. In response to ER stress, cells initiate an adaptive response named unfolded protein response (UPR), which reduces misfolded protein load. However, when cells undergo irreversible ER stress, UPR triggers apoptosis by upregulating an apoptotic effector named C/EBP-homologous protein (CHOP) [20,21,22].

Moreover, UPR has been identified to be associated with regulation of microRNA (miR) [23,24]. MicroRNA is composed of 20~22 nucleotide molecules that generally affect target mRNAs [25,26]. Among the many types of microRNAs, miR-216b is known to mediate the inhibition of progression and pathogenesis of liver and colorectal cancers [27,28]. In particular, CHOP has been involved in targeting post-transcriptional miR-216b, which reduce c-Jun expression and increase caspase-3, resulting in facilitating cell death [29].

To find anti-cancer effects of medical herbs against myeloid-originated hematological malignancies, a screening test was conducted with *Salvia miltiorrhiza* (SM), *Cnidium officinale* Makino (COM), *Achyranthes bidentata* Blume (ABB), *Eupolyphaga sinesis* Walker (ESW), and *Hirudo nipponica* Whitman (HNW) (Supplementary Figures S1 and S2). Among these medical herbs, SM and COM were evaluated to have apoptotic effects.

*Salvia miltiorrhiza* is a traditional medical herb that belongs to the family of Labiatae. It has been used for such clinical applications as activating blood circulation, removing blood stasis, and nourishing blood [30]. Previously, several studies have reported its biological anti-cancer effects in hematological malignancies through cytotoxic effects [31], suppression of Bcl-2 [32], and activation of Bax and caspase-3 [33]. However, the apoptotic effect of *Salvia miltiorrhiza* ethanol extract in myeloid-originated cancer cells, via regulation of miR-216b and ER stress, has not yet been elucidated. Thus, for this study, we used multiple myeloma cell line U266, myeloid leukemia cell line U937, and murine macrophage cell line Raw264.7. U266 cells are described to express a malignant disorder of differentiated human B cells. U937 represents myeloid leukemia and is known to differentiate into morphologically immature white blood cells.

In this study, the ER stress-mediated apoptotic effect of SM through miR-216b activation in myeloid-originated hematological malignancies cell lines has been studied.

## 2. Results

### 2.1. Salvia miltiorrhiza (SM) Suppresses the Growth of U266 and U937 Cells in a Concentration-Dependent Manner

**Figure 1:**
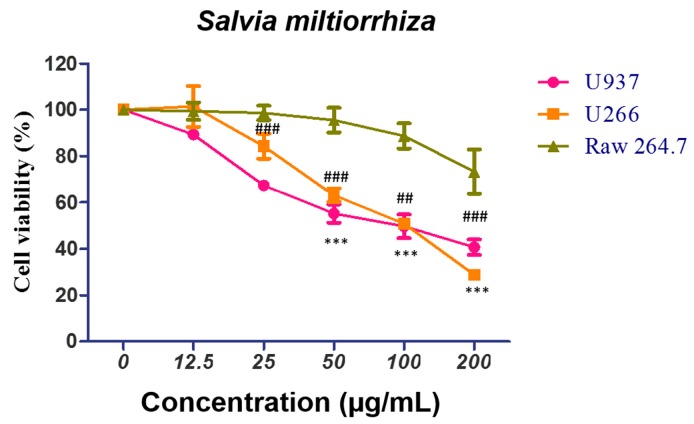
*Salvia miltiorrhiza* (SM) exerts a cytotoxic effect on U266 and U937 cells. Raw264.7 (murine macrophage), U266 (human multiple myeloma), and U937 (human myeloid leukemia) cells were grown in microplates (96 wells) at a density of 2 × 10^4^ cells/well. Those cell lines were treated with the indicated concentrations of SM (0, 12.5, 25, 50, 100, or 200 µg/mL) for 24 h. Cell viability was assessed by an EZ-cytox enhanced cell viability assay kit. Values represent the means of three experiments ± SD; ^##^, *p* < 0.01; ^###^, *p* < 0.001 versus an untreated control group (U937 cells); ***, *p* < 0.001 versus untreated control group (U266 cells).

To examine the cytotoxic effect of SM in U266 and U937 cells, an EZ-Cytox assay was conducted. Cells were treated with different concentrations of SM (12.5, 25, 50, 100, and 200 µg/mL) for 24 h. As shown in Figure 1, SM hampered the viability of U266 cells, with the death rates around 16% at a dose of 25 µg/mL, 37% at a dose of 50 µg/mL, and 50% at a dose of 100 µg/mL. Also, SM-treated U937 cancer cells showed cytotoxicity, with death rates of approximately 33% at a dose of around 25 µg/mL, 45% at a dose of 50 µg/mL, and 51% at a dose of 100 µg/mL. However, SM exhibited a lower cytotoxic effect on the normal macrophage cell line Raw264.7, with around 1% at a 25 µg/mL, 4% at a dose of 50 µg/mL, and 13% at a dose of 100 µg/mL.

### 2.2. SM Increases Reactive Oxygen Species (ROS) Generation and Cytotoxic Effect Is Dependent on ROS

**Figure 2:**
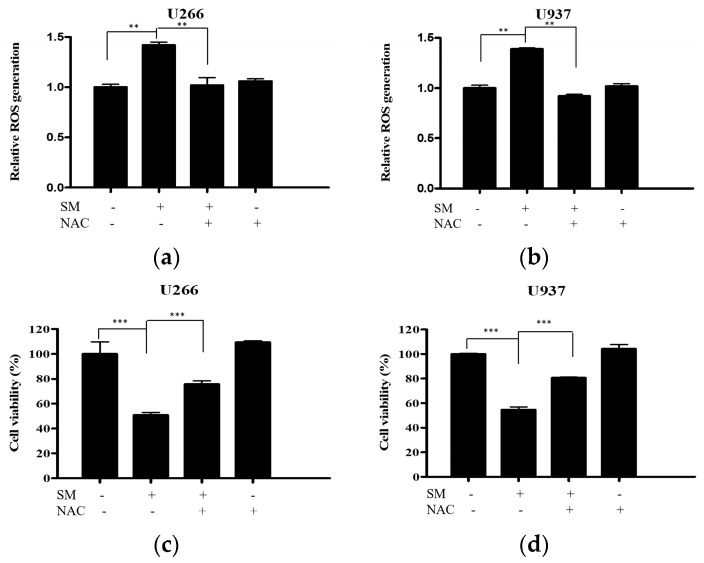
SM increases reactive oxygen species (ROS) production and ROS is required for an SM-induced cytotoxic effect on U266 and U937 cells. (**a**) U266 cells were treated with SM (50 µg/mL) for 24 h with or without pre-treatment of NAC (5 mM) for 1 h. ROS production was analyzed using a 2′,7′ -dichlorofluorescin diacetate (DCFDA) ROS detection assay kit. Values represent the means of three experiments ± SD; **, *p* < 0.01 versus the SM-only treated group; (**b**) U937 cells were processed under the same conditions; (**c**) U266 cells were treated with SM (50 µg/mL) for 24 h with or without pre-treatment of NAC (5 mM) for 1 h. Cell viability was determined by an EZ-cytox enhanced cell viability assay kit; (**d**) U937 cells were processed under the same conditions. Values represent the means of three experiments ± SD; ***, *p* < 0.001 versus an SM-only treated control.

To reveal the role of ROS in SM-induced apoptosis, ROS generation was measured. Cells were treated with 50 µg/mL of SM for 24 h. SM significantly increased the ROS production in U266 and U937 cells. The elevation of ROS generation is reversed by NAC pretreatment in both cells (Figure 2a,b). Furthermore, the decreased cell viability of SM-treated cells was recovered by NAC pretreatment (Figure 2c,d).

### 2.3. SM-Induced ER Stress Mediates Apoptosis

**Figure 3:**
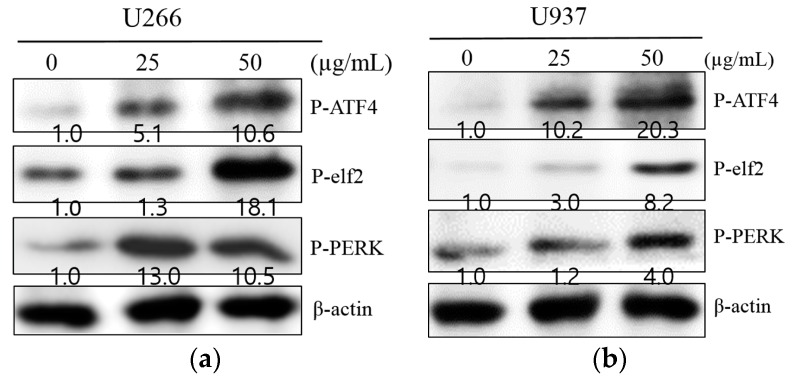
SM induces endoplasmic reticulum (ER) stress-related proteins in U266 and U937 cells. (**a**) U266 cells were treated with SM (25 or 50 μg/mL) for 24 h, and Western blot analysis was assayed for expression of P-PERK, P-eIF2α, P-ATF4, and β-actin; (**b**) U937 cells were experimented with under the same conditions.

**Figure 4:**
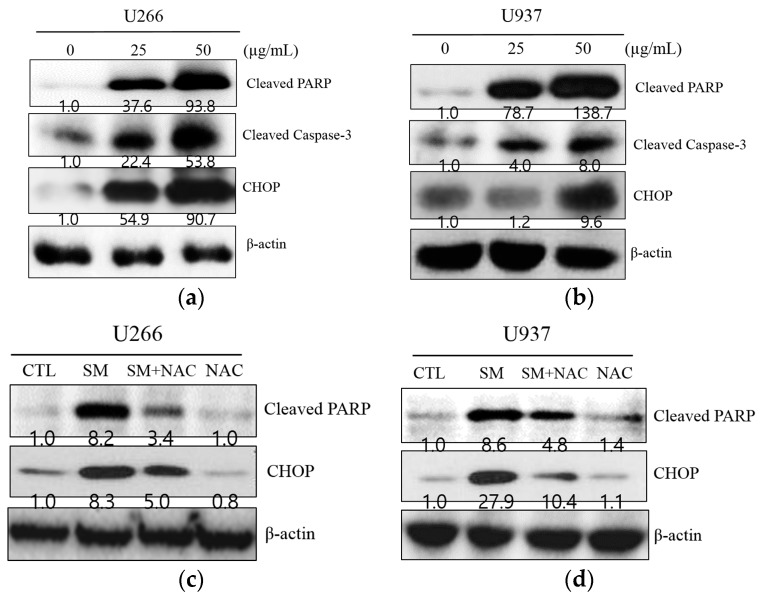
NAC reverses the expression of C/EBP homologous protein (CHOP) and cleavage of PARP in U266 and U937 cells. (**a**) U266 cells were treated with SM (25 or 50 μg/mL) for 24 h, and Western blot analysis was performed for cleaved PARP, cleaved caspase-3, and CHOP; (**b**) U937 cells were processed under the same conditions; (**c**) U266 cells were treated with SM (50 µg/mL) for 24 h with or without pre-treatment of NAC (5 mM) for 1 h. Cell lysates were prepared and subjected to Western blot analysis for CHOP and cleavage of PARP; (**d**) U937 cells were processed under the same conditions.

To investigate whether the anti-proliferative effect of SM is associated with apoptosis, Western blot analysis was performed. As illustrated in Figure 3a,b, SM increased ER stress-related proteins, such as p-ATF4, p-eIf2, p-PERK, and CHOP, in U266 and U937 cells. SM caused CHOP activation and PARP and caspase-3 cleavage in U266 and U937 cells at a concentration of 25 and 50 µg/mL (Figure 4a,b). To further substantiate the role of ROS in the apoptotic effect of SM, the expression of CHOP and cleaved PARP was measured without or with NAC. Cells were exposed to 25 or 50 µg/mL of SM for 24 h with/without NAC pretreatment. As shown in Figure 4c,d, SM activated CHOP and cleaved PARP. However, activated CHOP and PARP cleavage were reversed by NAC-pretreated U266 and U937 cells.

### 2.4. SM Increases Expression of miR-216b and Decreases Its Target, c-Jun, in U266 and U937 Cells

**Figure 5:**
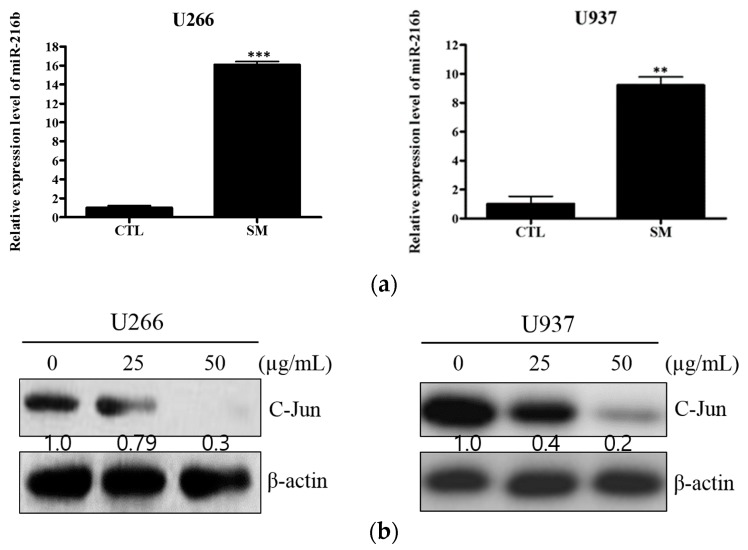
SM increases the expression of miR-216b and represses its target c-Jun. (**a**) In U266 and U937 cells, miR-216b levels were measured by a real-time PCR assay after 24 h of SM (50 µg/mL) treatment. Values represent the means of three experiments ± SD; **, *p* < 0.01; ***, *p* < 0.001 versus an untreated control; (**b**) U266 and U937 cells were treated with SM (25 or 50 μg/mL) for 24 h and Western blot analysis was assayed for expression of c-Jun and β-actin.

miR-216b is reported as a tumor suppressor miRNA that targets c-Jun. To elucidate the further mechanism of SM-induced apoptosis, real-time PCR and Western blot analysis were performed. As depicted in Figure 5a, the expression level of miR-216b was significantly increased in SM-treated U266 and U937 cells, by 9-fold and 16-fold, respectively. Also, the target protein of miR-216b, c-Jun, was notably hampered in SM-treated cells (Figure 5b).

### 2.5. Inhibition of miR-216b Reverses SM-Induced Apoptosis

**Figure 6:**
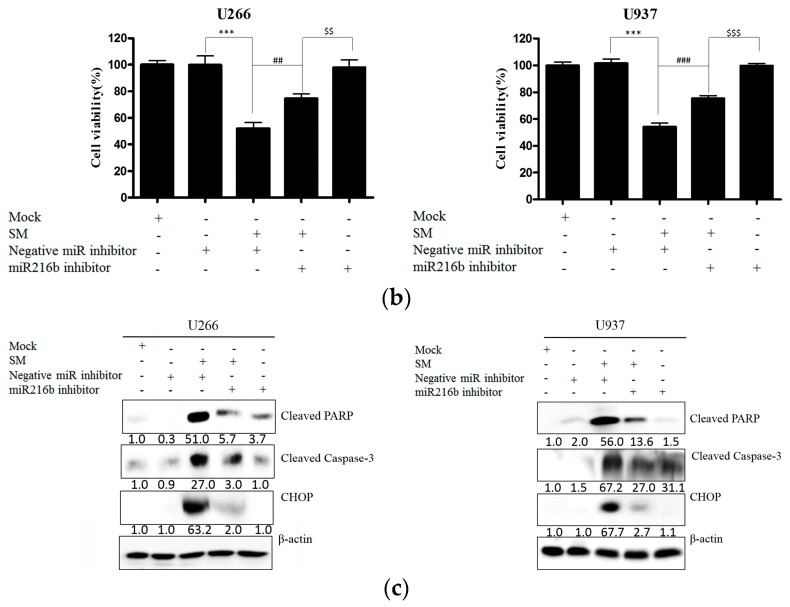
MiR-216b inhibition reversed the apoptotic effect of SM in U266 and U937 cells. (**a**) Cells were transfected with miRNA inhibitor negative control (Negative miR inhibitor) or miR-216b inhibitor and treated with SM (50 μg/mL) for 24 h. Real-time PCR for miR-216b expression was conducted. Values represent the means of three experiments ± SD; ***, *p* < 0.001 versus the miRNA inhibitor negative control group; ^##^, *p* < 0.01; ^###^, *p* < 0.001 versus miR-216b inhibitor-transfected and SM-treated group; ^$$$^, *p* < 0.001 versus miR-216b inhibitor-transfected group; (**b**) The cell viability assay was performed with miR-216b-inhibited and 50 μg/mL of SM-treated cells. Values represent the means of three experiments ± SD; ***, *p* < 0.001 versus the miRNA inhibitor negative control group; ^##^, *p* < 0.01; ^###^, *p* < 0.001 versus the miR-216b inhibitor-transfected and SM-treated group; ^$$^, *p* < 0.01; ^$$$^, *p* < 0.001 versus the miR-216b inhibitor-transfected group; (**c**) Cells were transfected with miRNA inhibitor negative control or miR-216b inhibitor and treated with 50 μg/mL of SM for 24 h. Western blot analysis was conducted for cleaved PARP, cleaved caspase-3, and CHOP. Mock, group treated with transfection reagent only; SM, SM-treated group.

To identify the role of miR-216b in SM-induced apoptosis, miR-216b was inhibited and real-time PCR for miR-216b and a cell viability assay and Western blot analysis for cleaved PARP, cleaved caspase-3, and CHOP were performed. As shown in Figure 6a, SM increased miR-216b in both U266 and U937 cells. Also, the cytotoxic effect of SM was found to be upregulated (Figure 6b). However, increased miR-216b was significantly suppressed by miR-216b inhibitor transfection in both U266 and U937. In line with these data, the cytotoxic effect of SM was found to be notably decreased by the miR-216b inhibition (Figure 6b). In addition, SM increased expression of cleaved PARP, cleaved caspase-3, and CHOP. In contrast, the cleaved PARP and caspase-3 and activated CHOP by SM treatment were attenuated by miR-216b inhibition (Figure 6c).

## 3. Discussion

Human multiple myeloma and myeloid leukemia are broadly representative types of myeloid-originated hematological malignancies. Multiple myeloma is a clonal proliferation of malignant plasma cells and human myeloid leukemia is an aberrant accumulation of immature white blood cells. Nearly all patients with myeloid-originated hematological malignancies succumb to a relapse and long-term survival is poor for most cases [34,35].

In the context of cancer therapy, several natural products have been discovered to possess anti-cancer effects, including cell cycle arrest, an anti-angiogenesis effect, and autophagy. [36,37,38]. Recent studies reported *Salvia miltiorrhiza*-induced anti-carcinogenic effects in multiple myeloma and human myeloid leukemia [30,39]. Further, in our previous article, we demonstrated that *Salvia miltiorrhiza* induces G2/M arrest in multiple myeloma [40] and autophagic cell death in multiple myeloma and human chronic myelogenous leukemia [31].

Traditional medicines have been used for thousands of years to treat all kinds of diseases, including cancer, in the world. In Korea, traditional medicine is still well-used under the supervision of the Ministry of Health and Welfare of Korea by licensed traditional doctors. There is a categorized list of herbs, named Hwal-Hyeol-Geo-Yeo (HHGY) herbs, including *Cnidium officinale* Makino (COM), *Salvia miltiorrhiza*, *Achyranthes bidentata* Blume (ABB), *Eupolyphaga sinesis* Walker (ESW), and *Hirudo nipponica* Whitman (HNW). They help smooth blood circulation and decrease the viscosity of blood by anti-thrombotic activities. Hematological cancer induces a hypercoagulability state of patients with at least one marker of thrombosis shown in 82.7% of patients, which increases morbidity and mortality [41]. In that respect, the HHGY herbs could be effective anti-multiple myeloma drug candidates. Thus, anti-MM effects and mechanisms of HHGY herbs were studied (Supplementary Figures S1 and S2). COM showed anti-MM properties via ROS generation-dependent ER stress-induced apoptosis similar to SM. However, there are differences in epigenetic mechanisms between COM and SM. COM suppressed one of the onco-miRNA, miR-211, which in turn targeted and repressed the CHOP [42]. SM induced apoptosis by miR-216b upregulation, which attenuated c-Jun expression.

*Salvia miltiorrhiza* has been applied in the treatment of blood-related diseases, including blood stasis, premenstrual syndrome, insomnia, and cancer. Its active compounds are tanshinone I, tanshinone IIA, cryptotanshinone, salvianolic acid B, protocatechuic aldehyde, etc. [43]. It has been reported to have an anti-inflammatory effect [44], an anxiolytic effect [45], and anti-cancer effects [46,47]. However, ER stress-mediated apoptotic effects of SM correlated with regulation of miR-216b have not been fully revealed yet. Therefore, we focused on finding anti-cancer effects of *Salvia miltiorrhiza* through CHOP-dependent miR-216b downstream using multiple myeloma cell line U266 and human myeloid leukemia cell line U937.

The endoplasmic reticulum (ER), where the initial steps for the maturation of protein are processed, is significantly responsible for the protein folding that is synthesized in the secretory pathway [48]. In the present study, an anti-proliferative effect of SM against U266 and U937 cells was identified (Figure 1). Also, *Salvia miltiorrhiza*-induced ROS, which is an ER stress factor, was demonstrated by pretreatment with NAC, an ROS scavenger (Figure 2). Furthermore, *Salvia miltiorrhiza*-induced ROS generation increased expression of CHOP and this was proven by pretreatment with NAC, an ROS scavenger (Figure 4c,d) These findings are in agreement with a previous report that suggested Tanshinone IIA, one of the phytocompounds of SM, increases ROS in ER-mediated apoptosis [49].

A massive accumulation of unfolded proteins is sensed by the ER stress sensors activate transcription factor 6 (ATF6), inositol-requiring protein 1 (IER1), and protein kinase RNA-like ER kinase (PERK). If ER stress is facilitated to the extent that UPR is unable to cope with unfolded protein accumulation, three branches of UPR, ATF6, IRE1, and PERK signaling, transduce an apoptotic downstream pathway [12].

Out of the three branches, PERK signaling in particular mediates phosphorylation of eukaryotic translation initiator factor 2α (eIF2α). Phosphorylation of eIF2α also allows for the expression of activate transcription factor 4 (ATF4), thereby leading to accumulation of the C/EBP-homologous protein (CHOP) involved in apoptosis [21]. In agreement with this, we found that SM triggered the PERK signaling pathway (Figure 3). Furthermore, we indicated that SM induced PERK-dependent CHOP expression and increased the apoptotic marker cleaved PARP, which is in accordance with previous studies (Figure 4) [50,51].

In addition, PERK has been reported to regulate directly miRNA expression, which shifts the balance between survival and cell death during ER stress [52]. Recently, miR-216b was shown to induce arrest in tumor growth [53,54]. A previous study indicated that PERK-signaling-dependent miR-216b is proven to be a direct transcriptional target of CHOP [29].

In fact, miR-216b, a post-transcriptional target of CHOP, suppresses c-Jun expression, which protects cells from apoptosis of tumor necrosis factor-α (TNF-α) [55,56]. In accordance with these previous reports, we observed that SM increased the expression level of miR-216b in both U266 and U937 cells and its role in SM-induced apoptosis was confirmed by cell viability assay and Western blot analysis with miR-216b inhibitor. Moreover, downregulation of c-Jun expression is observed (Figure 5b).

The downregulation of c-Jun is known to trigger caspase-3, ultimately inducing cleaved PARP, which increases susceptibility to apoptosis [29,57,58]. Indeed, production of caspase-3 and its substrate PARP were observed after treatment with SM (Figure 6).

Still, SM-mediated microRNA regulation in cell death pathways under ER stress needs further investigation. As noted above, SM and COM have similarities and differences in their efficacies. So, comparative experiments are needed with constituents of SM/COM and a combination treatment of SM and COM. Also, additional studies using various cancer cell lines, such as breast cancer, hepatocellular cancer, and rectal cancer, and animal studies are required to expand the therapeutic use of SM in cancer treatment.

## 4. Materials and Methods

### 4.1. Chemicals and Reagents

Dried *Salvia miltiorrhiza* (SM, Beijing, China) (1 kg) was extracted with 99.9% ethyl alcohol (Duksan, Gyeonggi-do, Korea) for 48 h at room temperature. The solution was filtered and evaporated using a Yamato Vacuum rotary evaporator (EYELA, Yamato, Tokyo, Japan) at 40 °C and lyophilized at −80 °C in a vacuum freeze-dryer (EYELA, Yamato, Tokyo, Japan) to yield 40.319 g of crude extract. The lyophilized *Salvia miltiorrhiza* extract was then made into a powder form according to a previously described method [59]. It was dissolved in dimethyl sulfoxide (Duksan, Gyeonggi-do, Korea) as a stock solution (200 mM) and then stored at −20 °C. Its main constituents were previously reported to be cryptotanshinone, tanshinone I, and tanshinone IIA [60].

### 4.2. Cell Culture

Human multiple myeloma U266 cells were obtained from the American Type Culture Collection (ATCC, Manassas, VA, USA). Human myeloid leukemia U937 cells and mouse macrophage Raw264.7 cells were obtained from the KCLB (Korean cell line Bank, Seoul, Korea). They were grown in RPMI 1640 (Welgene, Daegu, Korea) medium supplemented with 10% fetal bovine serum (FBS) and 1% penicillin/streptomycin at 37 °C in a humidified incubator under 5% CO_2_.

### 4.3. Cytotoxicity Assay

The cell viability of SM was measured by an EZ-cytox cell viability assay kit (DoGen, Seoul, Korea). U266 and U937 cells were seeded onto 96-well microplates at a density of 2 × 10^4^ cells per well and treated with various concentrations of SM (0, 12.5, 25, 50, 100, or 200 µg/mL) for 24 h with or without pre-treatment of NAC (5 mM) for 1 h. Then, a volume of 10 µL of EZ-cytox kit solution was added to each well and incubated for another 2 h at 37 °C. Optical density (OD) was calculated using a microplate reader (Bio-Rad, Hercules, CA, USA) at 450 nm. Cell viability was shown as a percentage of the absorbance present in the SM-treated group compared with control cells.

### 4.4. Western Blot Analysis

U266 and U937 cells (1 × 10^6^ cells/mL) were treated with indicated concentrations of SM (25 or 50 µg/mL) for 24 h. Then, the cells were lysed with RIPA buffer (1 M EDTA, 1 mM Na_3_VO_4_, 1 mM NaF, 50 mM Tris-HCl, pH 7.4, 150 mM NaCl, 1% NP-40, 0.25% sodium deoxycholic acid) containing a protease inhibitors cocktail (Amresco, Solon, OH, USA). The protein supernatant was collected and quantified for protein concentration by using an RC DC protein assay kit II (Bio-Rad, Hercules, CA, USA). The proteins (30 µg) were separated via SDS-polyacrylamide gel electrophoresis and transferred to nitrocellulose membranes that were blocked with 5% skim milk in Tris-buffered saline with 0.05% Tween 20 and incubated with the required antibodies. Primary antibodies, including cleaved PARP (1:1000, 89 kDa), CHOP (1:500, 27 kDa), P-eIF2α (1:500, 38 kDa) (Cell Signaling, Beverly, MA, USA), P-ATF4 (1:500, 39 kDa) (Thermo Fisher, Waltham, MA, USA), P-PERK (1:500, 125 kDa) (Thermo Fisher, Waltham, MA, USA), c-Jun (1:1000, 39 kDa), and β-actin (1:1000, 43 kDa) (Santa Cruz, Dallas, TX, USA), were used at a 1:500~1000 dilution (5% bovine serum albumin) and secondary antibodies at a 1:1000 dilution (5% skim milk) (Santa Cruz, Dallas, TX, USA). After detected protein bands were visualized by Hybond ECL (Amersham Pharmacia, Piscataway, NJ, USA), the blots were scanned.

### 4.5. Measurement of ROS Generation

U266 and U937 cells were seeded onto 96-well microplates at a final concentration of 1 × 10^5^ cells per well and treated with SM (50 µg/mL) for 24 h with or without pre-treatment of NAC (5 mM) for 1 h. After staining the cells in 2′,7′-dichlorofluorescin diacetate (DCFDA) Cellular Reactive Oxygen Species Detection Assay Kit (Abcam, Cambridge, UK) Solution (20 μM), they were incubated at 37 °C for 30 min in the dark. Plate measurement was performed on a fluorescence plate reader (Bio-Rad, Hercules, CA, USA) at Ex/Em = 485/535 nm in end point mode in the presence of compounds, media, or buffer. An identical untreated control and an NAC-only treated group were used on a screening test of *Salvia miltiorrhiza* and *Cnidium officinale* Makino.

### 4.6. Real-Time Polymerase Chain Reaction

U266 and U937 cells (2 × 10^5^ cells/mL) were treated with indicated concentrations of SM (25 or 50 µg/mL) for 24 h. Afterward, total RNA was extracted from individual SM-treated samples using the TRIzol extraction method as described by the manufacturer (Invitrogen, Carlsbad, CA, USA). RNA was reverse transcribed using a Mir-X miRNA First-Strand Synthesis Kit (Takara Bio, Mountain View, CA, USA) according to the manufacturer’s protocol. Quantitative PCR was conducted using a standard protocol from the SYBR Premix Ex Taq II (Takara Bio, Mountain View, CA, USA). Primer sequences (human) were as follows: mature hsa-miR-216b Forward 5′-AAATCTCTGCAGGCAAATGTGA-3′ and U6 primers using (Cat. #638313, Takara Bio, Mountain View, CA, USA).

### 4.7. SiRNA Transfection

The siRNA transfection was performed using a TransITX2^®^ Dynamic Delivery System kit (Mirus, MD, USA). U266 and U937 cells were seeded onto 6-well plates at a density of 2.5~5.0 × 10^5^ cells per well and the cell cultures were incubated overnight. Opti-MEM I Reduced-Serum medium was added to 25 nM siRNA and 7.5 µL *Trans*IT-X2 and gently mixed then incubated at room temperature for 15–30 min. Then, U266 and U937 cells were exposed to SM (50 µg/mL) for 24 h. Primer sequences (human) were as follows: Anti-has-miR-216b:5′-Mu/ZEN/mCmAmCmAmUmUmUmGmCmCmUmGmCmAmGmAmGmAmUmU/3ZEN/-3′. miRNA inhibitor negative control #1 was purchased (Cat. #SMC-2101, BIONEER, Seoul, Korea).

### 4.8. Statistical Analysis

Data were expressed as mean ± SD from at least three independent experiments. Statistical evaluation of the data was performed using one-way analysis of variance (ANOVA) tests and Student’s *t* test for independent or correlated samples. A difference was considered significant if the p value was less than 0.05.

## 5. Conclusion

The results herein indicate that SM induces ER stress by producing ROS and the activated CHOP expression is followed by an elevated level of miR-216b expression in both U266 and U937. Also, SM reduced protein expression of c-Jun, which is a target of miR-216b, correlating to induction of cleavage of caspase-3 and PARP. However, the SM-treated U266 cells showed a more powerful effect on cytotoxicity than SM-treated U937 cells. The difference might be due to a different ROS generation level and a different miR-216b modification level.

## Supplementary Materials

Supplementary materials can be found at http://www.mdpi.com/1422-0067/19/4/1240/s1.
