# Supplementary material for: Activation of ER Stress-Dependent miR-216b Has a Critical Role in Salvia miltiorrhiza Ethanol-Extract-Induced Apoptosis in U266 and U937 Cells
Source: Int J Mol Sci. 2018 Apr 19;19(4):1240. doi: 10.3390/ijms19041240 (PMC5979365; doi:10.3390/ijms19041240)
Supplement: Supplementary file 1 [file ijms-19-01240-s001.zip › ijms-281901-supplementary.pdf]

Activation of ER Stress-Dependent miR-216b Has a Critical Role in *Salvia miltiorrhiza* Ethanol-Extract-Induced Apoptosis in U266 and U937 Cells

Changmin Kim [1,†], Hyo-Sook Song [2,†], Hojung Park [1,†] and Bonglee Kim [1,*]

[1] Department of Pathology, College of Korean Medicine, Graduate School, Kyung Hee University, 1 Hoegi-dong, Dongdaemun-gu, Seoul 130-701, Korea; ckdals4302@khu.ac.kr (C.K.); rosapark93@khu.ac.kr (H.P.)

[2] Department of Science in Korean Medicine, College of Korean Medicine, Graduate School, Kyung Hee University, 1 Hoegi-dong, Dongdaemun-gu, Seoul 130-701, Korea; shs331@khu.ac.kr

* Correspondence: bongleekim@khu.ac.kr; Tel.: +82-2-961-9217

† These authors contributed equally to this work.

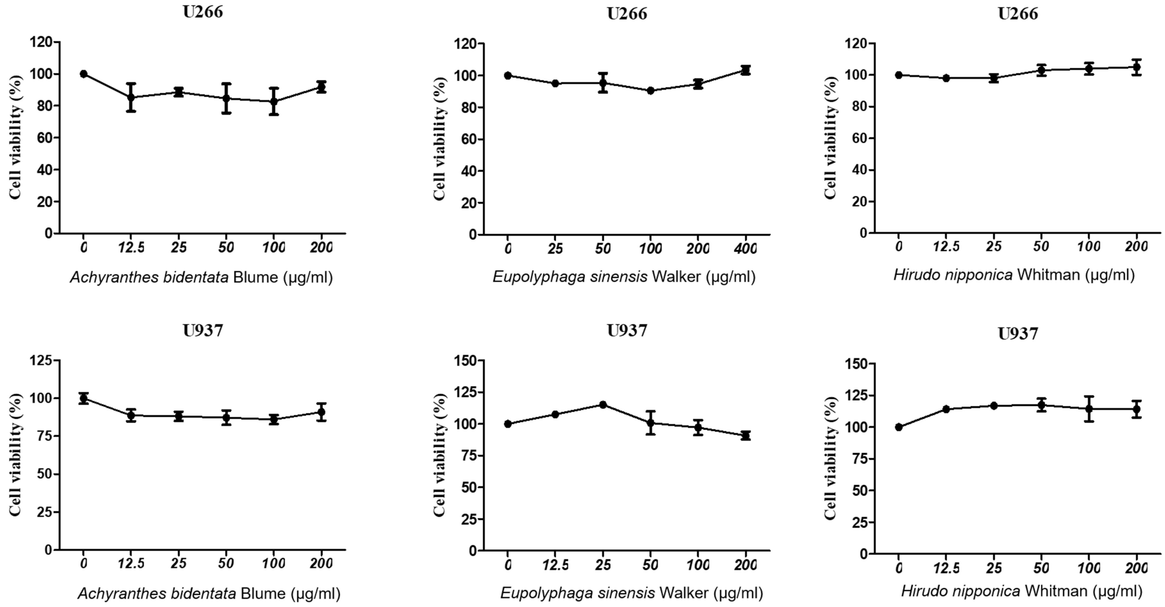

Supplementary Figure S1. Cytotoxic effect of *Achyranthes bidentata* Blume (ABB), *Eupolyphaga sinesis* Walker (ESW) and *Hirudo nipponica* Whitman (HNW) in U937 and U266 cells. Cells were seeded into 96 well microplates at a density of 2 x 10^4 cells/well and treated with various concentrations of ABB, ESW and HNW (0, 12.5, 25, 50, 100 or 200 μg/ml) for 24 h. Cell viability was measured by EZ-cytox Enhanced cell viability assay kit. Values represent the means of 3 experiments ± SD.

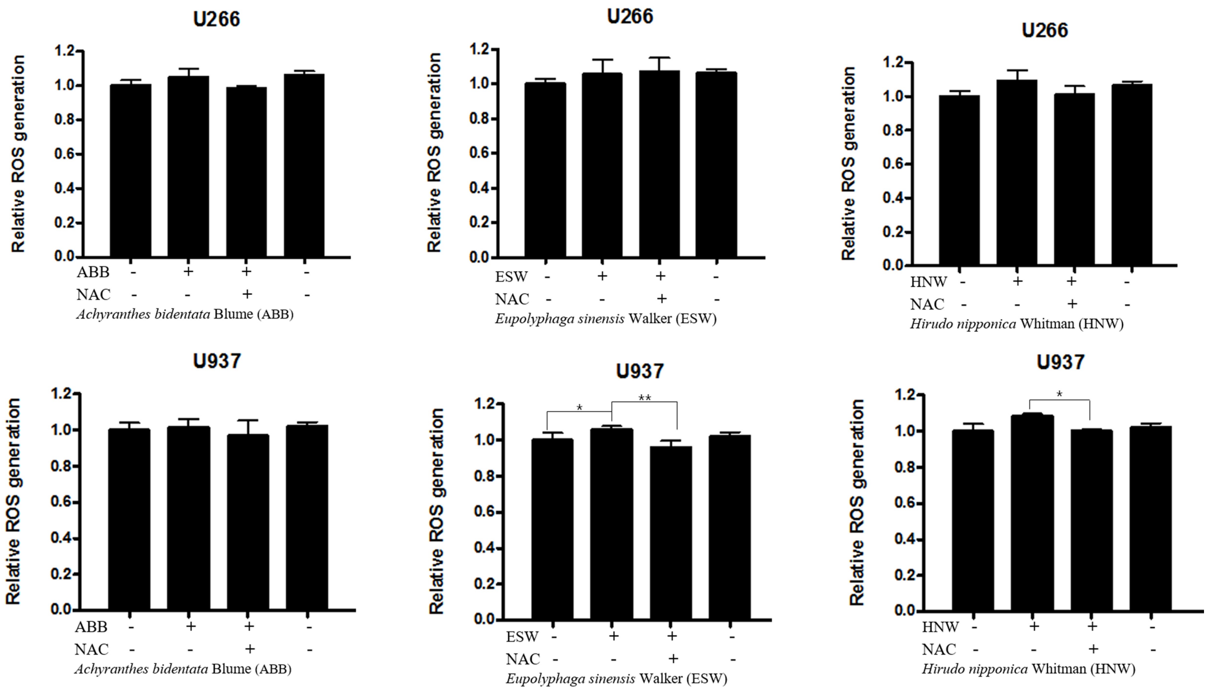

Supplementary Figure S2. The effect of COM on ROS production in *Achyranthes bidentata* Blume (ABB), *Eupolyphaga sinesis* Walker (ESW), *Hirudo nipponica* Whitman (HNW) treated U937 and U266 cells. Cells were treated with indicated extracts (80 μg/ml) for 24 h with or without pre-treatment of NAC (5 mM) for 1 h. ROS production was determined by cellular reactive oxygen species detection assay kit. Values represent the means of 3 experiments ± SD.
